# Alterations in Gene Expression of Components of the Renin-Angiotensin System and Its Related Enzymes in Lung Cancer

**DOI:** 10.1155/2017/6914976

**Published:** 2017-07-16

**Authors:** Benjamin Goldstein, Malav Trivedi, Robert C. Speth

**Affiliations:** ^1^Cooper City High School, Cooper City, FL, USA; ^2^College of Pharmacy, Nova Southeastern University, Fort Lauderdale, FL, USA

## Abstract

**Objectives:**

The study assessed the existence and significance of associations between the expression of fifteen renin-angiotensin system component genes and lung adenocarcinoma.

**Materials and Methods:**

NCBI's built-in statistical tool, GEO2R, was used to calculate Student's *t*-tests for the associations found in a DNA expression study of adenocarcinoma and matched healthy lung tissue samples. The raw data was processed with GeneSpring™ and then used to generate figures with and without Sidak's multiple comparison correction.

**Results:**

Ten genes were found to be significantly associated with adenocarcinoma. Seven of these associations remained statistically significant after correction for multiple comparisons. Notably, AGTR2, which encodes the AT_2_ angiotensin II receptor subtype, was significantly underexpressed in adenocarcinoma tissue (*p* < 0.01). AGTR1, ACE, ENPEP, MME, and PRCP, which encode the AT_1_ angiotensin II receptor, angiotensin-converting enzyme, aminopeptidase N, neprilysin, and prolylcarboxypeptidase, respectively, were also underexpressed. AGT, which encodes angiotensinogen, the angiotensin peptide precursor, was overexpressed in adenocarcinoma tissue.

**Conclusion:**

The results suggest an association between the expression of the genes for renin-angiotensin system-related proteins and adenocarcinoma. While further research is necessary to conclusively demonstrate a link between the renin-angiotensin system and lung cancers, the results suggest that the renin-angiotensin system plays a role in the pathology of adenocarcinoma.

## 1. Introduction

**Figure 1:**
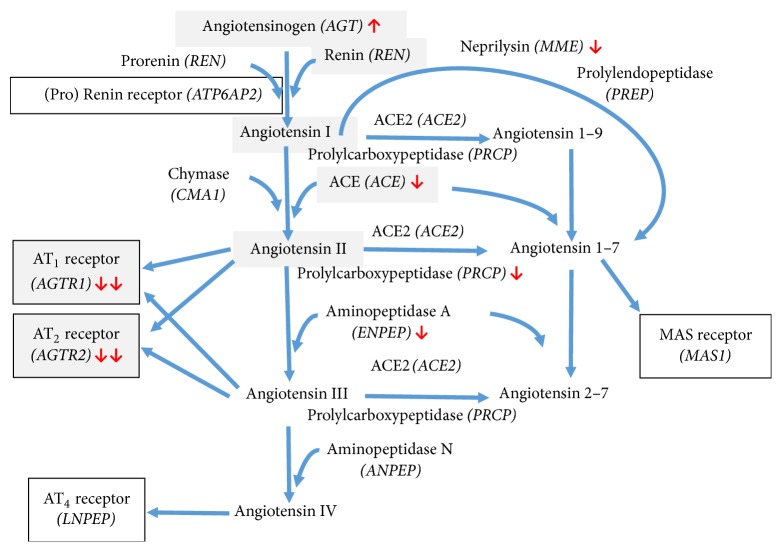
*Pathways of the renin-angiotensin system (RAS) cascade*. The classical RAS cascade is shown with light shading and includes the two major angiotensin II receptor subtypes, AT_1_ and AT_2_. Nonclassical components of the RAS cascade would include the ability of the (pro)renin receptor to activate prorenin, as well as enhance the activity of renin to metabolize angiotensinogen to Ang I, the ability of chymase to convert Ang I to Ang II, metabolism of Ang II to III and III to IV by aminopeptidases A and N, respectively, and the novel AT_4_ receptor for Ang IV which is also known as insulin-regulated aminopeptidase. In addition, there is the ACE2/Ang 1–7/mas axis of the RAS which is considered to be a counterregulator of the actions of the classical RAS. This includes the enzyme angiotensin-converting enzyme 2 (ACE2) which primarily forms Ang 1–7 from Ang II, but can also form Ang 1–9 from Ang I, prolylcarboxypeptidase which also forms Ang 1–7 from Ang II, and the enzymes neprilysin and prolylendopeptidase which forms Ang 1–7 directly from Ang I, and MAS, the putative receptor for Ang 1–7. The names of the genes that encode the various proteins of the RAS and related proteins are shown in italics after the protein names.

Lung cancer continues to be the leading cause of cancer deaths, and lung adenocarcinomas account for approximately 40% of all lung cancers (https://www.cancer.org/cancer/lungcancer-non-smallcell/index (accessed 8/31/16)). To assess factors associated with lung adenocarcinomas, a gene expression study comparing normal lung tissue and lung tumor was undertaken using the HG-U133A Affymetrix gene chip [1]. While such associations do not establish causation, they characterize the underlying molecular marks or tumors and identify potential target sites for treatments. To ascertain if the renin-angiotensin system (RAS) is altered in lung adenocarcinomas we evaluated the expression of 15 genes of the classical RAS (Figure 1) and extended components of the RAS as reported in a previous study [2].

The RAS (Table 1) functions primarily to maintain fluid and electrolyte homeostasis and regulate the cardiovascular system; however, the RAS is pleiotropic and is now recognized to have effects that extend beyond its primary functions. For example, Angiotensin II (Ang II), the primary hormone of the RAS, promotes the proliferation of mouse fibroblast 3T3 cells (but not SV40 transformed 3T3 cells in culture) [3], DNA synthesis and proliferation of adrenal cortical cells in culture [4], and the hypertrophic and hyperplastic growth of vascular smooth muscle cells [5]. Both myointimal hyperplasia and vascular smooth muscle DNA synthesis are increased in rats infused with Ang II [6]. All these proliferation-promoting effects of Ang II suggest that the RAS might play a critical role in cancer [7–10].

Canonically, most studies suggest that Ang II acting via the AT_1_ receptor can promote cancer by a variety of mechanisms. Ang II is angiogenic [11, 12] and can promote blood flow to tumors by stimulating vascular endothelial growth factor (VEGF) release; see reviews [7, 13]. Moreover, the tumor vessels are less responsive to Ang II such that the systemic vasoconstrictor actions of Ang II shunt blood to the tumor vasculature [14, 15]. Stimulation of the AT_1_ receptor by Ang II transactivates the EGF receptor [16, 17], a receptor that is overexpressed in many cancers [18, 19]. Activation of the AT_1_ receptor promotes the release of a host of growth factors that also promote cell proliferation [7]. Additionally, in several animal models of cancer, both angiotensin-converting enzyme (ACE) inhibitors and AT_1_ angiotensin receptor subtype blockers (ARBs) inhibit tumor growth [7].

Since both ACE inhibitors and ARBs are widely used antihypertensive agents, meta-analyses have been conducted to identify any correlation for cancer incidence in patients taking these drugs and/or the effects of these drugs on cancer risk. Surprisingly, there is not universal agreement on the effects of ACE inhibitors and ARBs on cancer incidence. For example, a meta-analysis reported in 2010 [20] found a slight increase in cancer risk in individuals that were being treated with ARBs. However, a subsequent meta-analysis published the following year [21] found no association between the use of ARBs and cancer risk. However, they did observe an increase in cancer risk in patients taking both an ARB and an ACE inhibitor. Of note the previous study [20] compared ARB + ACE inhibitor treated groups compared to ACE inhibitor only groups to show the association of ARB use with increased cancer risk. An epidemiological study of ACE inhibitor and/or ARB usage reported an increased risk of cancer deaths [22]; however, a study from the United States Food and Drug Administration [23] found no evidence for an increased cancer risk from ARBs. A recent meta-analysis of RAS blockade with ACE inhibitors and/or ARBs suggested that RAS blockade reduces cancer risk [24]. Another recent meta-analysis focusing on lung cancer risk in patients taking ARBs indicated that ARBs reduce lung cancer risk [25] with all but one [26] of 6 studies showing a reduced risk of cancer with 2 of the studies showing a significant reduction in lung cancer risk on their own [27, 28]. Thus the preponderance of clinical data suggests that ARBs reduce lung cancer risk but that there may be individual or population differences in lung cancer risks associated with RAS blockers. To further explore this putative relationship this study investigates changes in gene expression of RAS and related protein in a population of lung adenocarcinoma patients [1]. This could help to identify specific genes that could be investigated further to determine the exact role of RAS and extended RAS pathway genes in lung adenocarcinomas.

## 2. Methods and Materials

The study is an in silico analysis of the expression of renin-angiotensin system genes in connection with lung cancer. A total of 15 renin-angiotensin system related genes were studied (see Table 1 and Figure 1). The gene expression analysis was conducted on data sourced from NCBI's GEO database as log2 values. The specific dataset used, GSE10072, profiled the gene expression of lung adenocarcinoma tissue samples and paired noncancerous tissue samples [1].

The dataset was analyzed through the GEO database's integrated statistics utility, GEO2R, in addition to PRISM™ (v.6), GeneSpring (v.13.1), and Microsoft Excel. More specifically, GEO2R calculated *p* values in order to identify significant differences in gene expression while GeneSpring extracted and normalized the raw expression data using provided algorithms to allow for further statistical calculations using PRISM (Graphpad Software), which was also used to generate graphs and other figures from tables of raw data created in Excel. One value for ANPEP in the tumor group was excluded based upon the criterion of being more than 5 standard deviations from the mean.

Since the number of samples in this specific data set differed between the tumor (*n* = 57) and the normal tissue (*n* = 49) groups, comparisons were made using an unpaired Student's *t*-test with Welch's correction for heterogeneity of variance. Data is presented with and without Sidak's correction for multiple comparisons: *α* = 0.05, *α*_*C*_ = 1 − (1 − 0.05)^(1/15)^ (15 genes were studied) to provide a relative estimate of significance (Table 2). Percent changes in gene expression are reported as numerical values, and the analysis of numerical values is shown in the supplementary data section (Figure S1 in Supplementary Material available online at https://doi.org/10.1155/2017/6914976).

## 3. Results

**Figure 2:**
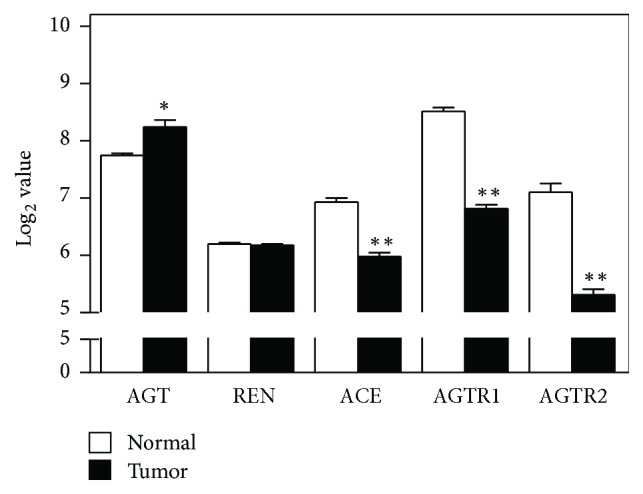
*Expression of genes of proteins that comprise the classical renin-angiotensin system*. Gene expression in normal lung tissue (open bars *n* = 49) and lung tumor tissue (solid bars, *n* = 58) is expressed as Log2 values. AGT encodes angiotensinogen, REN encodes renin, ACE encodes angiotensin-converting enzyme, AGTR1 encodes the AT_1_ Ang II receptor subtype, and AGTR2 encodes the AT_2_ Ang II receptor subtype. ^*∗*^*p* < 0.05 by unpaired *t* test with Welch's correction for heterogeneity of variance and Sidak's correction for multiple comparisons, ^*∗∗*^*p* < 0.01 by unpaired *t* test with Welch's correction for heterogeneity of variance and Sidak's correction for multiple comparisons.

There were a number of profound differences in the expression of the genes encoding the proteins comprising and closely associated with the renin-angiotensin system (RAS), between normal lung tissue and the lung tumor tissue (Figures 1 and 2 and Table 2). Additionally, there were substantial differences in the level of expression of the mRNA for the different proteins.

### 3.1. Classical RAS Pathway

Among the proteins that comprise the classical RAS, the gene for the AT_1_ subtype of the Ang II receptor was the most highly expressed in normal lung tissue (Figures 2 and S1, and Table 2). The mRNA for the other major Ang II receptor subtype AT_2_ was expressed at less than half the intensity as the AT_1_ receptor subtype. The gene expression for both Ang II receptor subtypes was dramatically reduced, 69 and 74%, respectively, (Figure S1), for AT_1_ and AT_2_ in the lung tumor tissue (*p* < 0.01, corrected for multiple comparisons, Table 2). The AGT gene which encodes angiotensinogen was also highly expressed in the normal lung tissue, and its mRNA was nearly doubled in the lung tumor tissue (*p* < 0.05, corrected for multiple comparisons Table 2). ACE, the gene that encodes the enzyme that converts the inactive precursor angiotensin I (Ang I) to the active hormone, Ang II, was expressed at a lower level and its expression was reduced by about half in the lung tumor tissue (*p* < 0.01 corrected for multiple comparisons Table 2). REN, the gene for renin, the enzyme which cleaves angiotensinogen to make Ang I, was the least highly expressed gene of the classical RAS proteins and its expression level was unchanged in the lung tumor tissue.

### 3.2. Extended RAS Pathway

**Figure 3:**
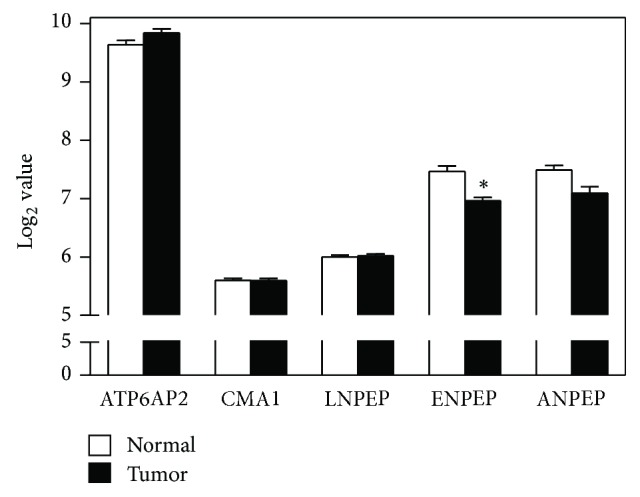
*Expression of genes of proteins that directly interact with renin-angiotensin system*. These genes encode proteins that can alter the function of RAS but have many other functions. ATP6AP2 encodes the prorenin receptor (ATPase H(+)-transporting accessory protein 2), CMA1 encodes chymase, LNPEP encodes the Ang IV (AT_4_) receptor (insulin-regulated aminopeptidase), ENPEP encodes aminopeptidase A, and ANPEP encodes aminopeptidase N. One value for ANPEP was excluded from the data analysis because it was >3 standard deviations apart from the rest of the dataset. ^*∗*^*p* < 0.05 by unpaired *t* test with Welch's correction for heterogeneity of variance and Sidak's correction for multiple comparisons.

Genes of the proteins that have ancillary interactions with the RAS also showed a considerable variation in expression level in normal lung tissue, and the expression of several of these genes were altered in the lung tumor tissue (Figures 3 and S1 and Table 2). ATP6AP2 which encodes the (pro)renin receptor, but which may have a more important cellular function in promoting hydrogen ion transport and Wnt signaling via the frizzled receptor [29] was the most highly expressed gene in the normal lung tissue. There was a small, 17%, increase in ATP6AP2 expression (Figure S1) that was not statistically significant after correction for multiple comparisons. REN expression in lung tumor tissue was also 17% higher than in normal lung tissue (Figure S1) and also was not statistically significant after correction for multiple comparisons (Table 2). CMA1, the gene for chymase, which can facilitate the RAS by converting Ang I to Ang II, was expressed at a low level and its expression did not differ between normal lung tissue and lung tumor tissue. LNPEP, the gene which encodes the AT_4_ receptor, but which may have a more important cellular function as insulin-regulated aminopeptidase [30], was also expressed at a low level and its expression did not differ between normal lung tissue and lung tumor tissue.

Expression of ENPEP, the gene which encodes aminopeptidase A, a major Ang II metabolizing enzyme, was expressed at a moderate level in normal lung tissue with a statistically significant (*p* < 0.001, corrected for multiple comparisons, Table 2) 32% reduction in its expression in lung tumor tissue (Figure S1). ANPEP, the gene which encodes aminopeptidase N, a major Ang III metabolizing aminopeptidase, was also expressed at a moderate level in normal lung tissue and at a slightly lower level in lung tumor tissue, but did not retain statistical significance (*p* = 0.054) after correction for multiple comparisons (Table 2).

### 3.3. ACE2/Ang 1–7/MAS Axis of the RAS

**Figure 4:**
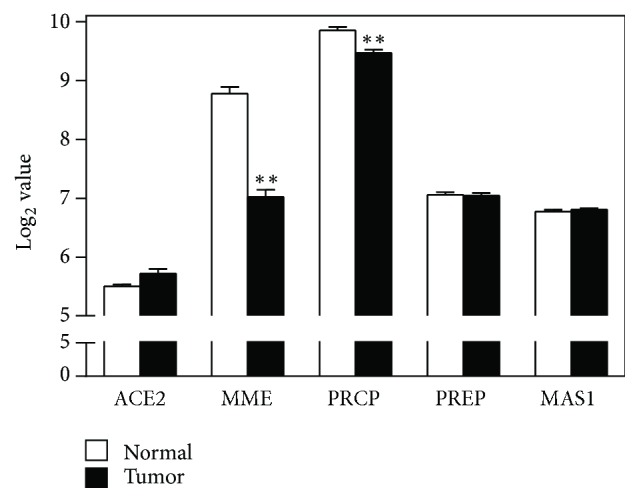
*Expression of genes of ACE2/Ang 1–7/MAS axis of the renin-angiotensin system*. Expression of genes encoding proteins of the ACE2/Ang 1–7/MAS axis of the RAS which is an arm of the RAS that generally counteracts the actions of the classical RAS. ACE2 encodes angiotensin-converting enzyme-2, MME encodes neprilysin (neutral endopeptidase), PRCP encodes prolylcarboxypeptidase, PREP encodes prolylendopeptidase, and MAS1 encodes MAS (Ang 1–7 receptor). ^*∗∗*^*p* < 0.01 by unpaired *t* test with Welch's correction for heterogeneity of variance and Sidak's correction for multiple comparisons.

The genes for the primary component proteins of the ACE2/Ang 1–7/MAS axis of the RAS, ACE2, and MAS were expressed at low levels in normal lung tissue and expression levels did not differ between normal lung tissue and lung tumor tissue (Figures 4 and S1 and Table 2). However, the genes for two enzymes that have ancillary functions associated with the ACE2/Ang 1–7/MAS axis; MME and PRCP, which encode neprilysin and prolyl carboxypeptidase, respectively, were highly expressed in normal lung tissue. Their expression was significantly decreased in lung tumor tissue (*p* < 0.01, corrected for multiple comparisons, Table 2) by 66% and 23%, respectively (Figure S1). PREP, which encodes prolyl endopeptidase, another enzyme that can promote the functionality of the ACE2/Ang 1–7/MAS axis, was expressed at a moderate level in normal lung tissue, but its expression was unaltered in lung tumor tissue.

## 4. Discussion

In view of the potential involvement of the RAS in promoting lung as well as other cancers (see reviews [7, 8]) the extent and changes in the expression of genes encoding proteins of the RAS, as well as proteins whose actions include alteration of classical RAS activity, are of considerable interest. The high level of expression of the gene encoding the AT_1_ receptor in the lung suggests that Ang II acting upon the AT_1_ receptor plays an important role in lung function and cell growth. Consistent with an oncogenic activity of the RAS in lung, AT_1_ receptor inhibition decreases the metastasis of lung cancer cells [31]. See also reviews 7–9. An additional mechanism whereby the RAS could increase tumor growth by activating AT_1_ receptors is by selectively increasing blood flow in newly formed vessels of tumors which, in contrast to the systemic vasculature, do not constrict in response to Ang II [32]. Corollary to such a mechanism, inhibitors of Ang II formation, agents that increase metabolism of Ang II, and AT_1_ receptor blockers that lower BP may decrease tumor growth by disproportionately reducing blood flow to the tumor.

Also consistent with the ability of the AT_1_ receptor to promote cancer cell growth, the AT_1_ receptor can transactivate the EGF receptor, a known oncogenic stimulus [16]. However, the reduced expression of the AT_1_ receptor gene in lung tumor cells, suggests that AT_1_ receptor stimulation of the tumor cells may not be a driving force for their oncogenic activity. Conversely, the slightly greater reduction in AT_2_ receptor gene expression could contribute to the oncogenic activity of lung tumor cells by reducing the inhibitory actions on cell growth that Ang II causes by activating the AT_2_ receptor. Indeed, when AT_2_ receptors were overexpressed in lung adenocarcinoma cells, their growth was inhibited [33].

The increased expression of the precursor protein of the RAS, angiotensinogen, in lung tumor tissue suggests that there may be an increased activation of the RAS in lung tumor cells. Angiotensinogen can be rate limiting in the production of Ang II, although the concentrations of renin, prorenin, and the (pro)renin receptor are the primary determinants of the rate of formation of Ang I and Ang II. Of note, however, there is a non-RAS functionality of angiotensinogen to interact with DNA, in particular to reverse formation of carcinogenic DNA adducts generated by the contents of tobacco smoke [34]. In this case the increase in angiotensinogen gene expression in lung tumor cells could be a compensatory response of the cells to normalize their DNA by removing the adducts formed by tobacco smoke carcinogens. Additionally, angiotensinogen, as well as des-Angiotensin I angiotensinogen, has antiangiogenic effects and reduces metastasis of B16-F10 melanoma cells to the lungs of mice [35].

Consistent with the reduced expression of ACE in the lung tumor tissue, serum levels of ACE are decreased in lung cancer patients to a greater extent than in other cancers [36]. While ACE inhibitors are heavily used as blockers of the RAS actions on the AT_1_ receptor, ACE is not the rate limiting step in Ang II production. Moreover, Ang II can also be generated by chymase, and the mRNA for chymase was not altered in the lung tumor cells. Another consideration is that the mRNA transcribe from the gene ENPEP which encodes aminopeptidase A, also known as angiotensinase A, a major metabolic enzyme of Ang II, is reduced and this could indicate a prolongation of the stimulation of AT_1_ and AT_2_ receptors by Ang II. While Ang III the product of aminopeptidase A metabolism of Ang II is a full agonist at AT_1_ and AT_2_ receptors, it is known to be rapidly metabolized following its formation, such that any slowing of the conversion of Ang II to Ang III will increase the stimulation of AT_1_ and AT_2_ receptors. Moreover, early studies of the proliferative effects of Ang II suggested that Ang III lacked the ability to stimulate cell proliferation [37].

The low level and apparent lack of differential expression of the genes encoding components of the ACE2/Ang 1–7/MAS axis in normal lung tissue and lung tumor tissue are surprising in view of the large body of literature suggesting that the ACE2/Ang 1–7/MAS axis plays an important role in lung function [38], susceptibility to the SARS virus [39], and inhibition of angiogenesis in lung cancer [40]. The reductions in the high level of expression of the genes for two enzymes capable of cleaving Ang I and Ang II to form Ang 1–7; neprilysin and prolyl carboxypeptidase, respectively, may cause a reduction in the shunting of Ang I and Ang II away from their roles in the classical RAS and reduce the antagonistic action of the ACE2/Ang 1–7/MAS axis. This would promote the actions of Ang II at the AT_1_ receptor while simultaneously reducing the stimulation of the MAS receptor by Ang 1–7.

It is well established that not all cancers are the same and it is not surprising that alterations in the RAS in different cancers are not consistent. For example, a large-scale study of estrogen receptor-positive breast cancer tumors revealed an increase in AGTR1 mRNA expression [41], which is the opposite of what we observed in the lung cancer study from which this data is derived. Equally contradictory, AGTR1 as well as ACE mRNA expression is upregulated in hormone-independent prostate cancer [42].

While the changes observed in mRNA expression in some of the components of the RAS in lung adenoma tumor cells are substantial, there are three caveats that must be acknowledged. First, it is uncertain whether these changes in mRNA are contributing to the phenotypic changes in lung cells to become cancer cells, or if they are a result of the phenotypic changes associated with the transformation of normal lung cells to cancer cells. Second, alterations in mRNA expression do not always reflect alterations in protein expression. To assess this second caveat it will be necessary to determine the level of expression of the proteins encoded by the mRNAs that showed significant changes. The third caveat relates to possible changes in signal transduction by the AT_1_, AT_2_, and MAS receptors. It is possible that signal transduction in response to activation of these receptors by Ang II, Ang III, and Ang 1–7 could be increased or decreased. It is known that the AT_1_ receptor transduces responses to Ang II via G protein-coupled and G protein independent pathways [43] and that peptide antagonists of the AT_1_ receptor can signal exclusively through the G protein independent pathway [44], so it is not unreasonable for AT_1_ receptor activation to have differential effects on cell proliferation due to differences in the efficiency of these two signaling pathways in cancer cells. While it would be of interest to assess the mRNA expression of these signal transduction proteins, it is not possible to specify that their changes are specifically linked to these angiotensin receptors since they mediate transduction by a variety of hormones, cytokines, and neurotransmitters.

## 5. Conclusion

The current study emphasizes the involvement of components of the RAS (both direct and extended) in the development and progression of lung cancer. In spite of the several limitations pertaining to the dataset, this study provides a novel therapeutic targeting modality for treatment of lung cancer. Moreover, the current study also provides a platform to mimic our approach of data mining previously conducted genome-wide expression studies to identify novel therapeutic targets for a variety of cancers and other diseases. This will decrease the cost burden of investigating new drug entities and possibly allow for the repurposing of already approved drugs.

## Supplementary Material

Figure S1: Expression of genes of proteins that comprise or interact with the renin-angiotensin system (RAS) in normal lung tissue (open bars *n* = 49) and lung tumor tissue (solid bars, *n* = 58) expressed as numeric values.

## Figures and Tables

**Table 1:** Proteins of the classical renin-angiotensin system (RAS) and proteins with RAS-related functions.

Name	Gene name	E.C. number	Function for RAS
Angiotensinogen	AGT	—	Precursor of Ang peptides
Renin	REN	3.4.23.15	Angiotensinogen to Ang I
Angiotensin-converting enzyme	ACE	3.4.15.1	Ang I to Ang II
Prorenin receptor	ATP6AP2	—	Activates prorenin, receptor for renin and prorenin
Chymase	CMA1	3.4.21.29	Ang I to Ang II
Neprilysin	MME	3.4.24.11	Ang I to Ang 1–7, Ang II to Ang 1–4 and 5–8
Prolylendopeptidase	PREP	3.4.21.26	Ang I to Ang 1–7, Ang II to Ang 1–7
Aminopeptidase A	ENPEP	3.4.11.7	Ang II to Ang III
Aminopeptidase N	ANPEP	3.4.11.2	Ang III to Ang IV
Angiotensin-converting enzyme 2	ACE2		Ang 1 to Ang 1–9, Ang II to Ang 1–7
Prolylcarboxypeptidase	PRCP	3.4.16.2	Ang II to Ang 1–7
AT_1_ Ang II receptor subtype	AGTR1	—	Ang II receptor subtype vasoconstriction, thirst
AT_2_ Ang II receptor subtype	AGTR2	—	Ang II receptor subtype generally opposes AT_1_ effects
MAS	MAS1	—	Ang 1–7 receptor
Insulin-regulated aminopeptidase		3.4.11.3	Ang IV receptor (AT_4_)

**Table 2:** Significance levels of differences in RAS and RAS-related protein gene expression in lung tumor and normal lung tissue based upon log2 values.

GENE	Protein	Change in expression in lung tumor	Significance level^*∗*^ (uncorrected for multiple comparisons)	Significance level (corrected for multiple comparisons)^*∗∗*^
AGT	Angiotensinogen	**↑**	*p* = 0.0004	*p* < 0.01
REN	Renin	**=**	NS *p* = 0.66	NS
ACE	Angiotensin-converting enzyme	**↓**	*p* < 0.0001	*p* < 0.01
AGTR1	AT_1_ Ang II receptor subtype	**↓**	*p* < 0.0001	*p* < 0.01
AGTR2	AT_2_ Ang II receptor subtype	**↓**	*p* < 0.0001	*p* < 0.01
ATP6AP2	Prorenin receptor	**↑**	*p* = 0.045	NS
CMA1	Chymase	**=**	NS *p* = 0.89	NS
LNPEP	Ang IV receptor, insulin-regulated aminopeptidase	**=**	NS *p* = 0.76	NS
ENPEP	Aminopeptidase A	**↓**	*p* < 0.0001	*p* < 0.01
ANPEP	Aminopeptidase N	**↓**	*p* = 0.0037	NS (*p* = 0.054)
ACE2	Angiotensin-converting enzyme-2	**↑**	*p* = 0.0087	NS
MME	Neprilysin	**↓**	*p* < 0.0001	*p* < 0.01
PRCP	Prolylcarboxypeptidase	**↓**	*p* = 0.0001	*p* < 0.01
PREP	Prolylendopeptidase	**=**	NS *p* = 0.83	NS
MAS1	Mas, Ang 1–7 receptor	**=**	NS *p* = 0.35	NS

^*∗*^Unpaired *t* test with Welch's correction; ^*∗∗*^Sidak's correction: (1 − (1 − .05)^1/15^) or (1 − (1 − .01)^1/15^) or (1 − (1 − .001)^1/15^; NS is nonsignificant.
