# Supplementary material for: Alterations in Gene Expression of Components of the Renin-Angiotensin System and Its Related Enzymes in Lung Cancer
Source: Lung Cancer Int. 2017 Jul 16;2017:6914976. doi: 10.1155/2017/6914976 (PMC5534309; doi:10.1155/2017/6914976)
Supplement: Supplementary file 1 — Figure S1: Expression of genes of proteins that comprise or interact with the renin-angiotensin system (RAS) in normal lung tissue (open bars n = 49) and lung tumor tissue (solid bars, n = 58) expressed as numeric values. [file 6914976.f1.doc]

**Supplementary Material**

**Figure S1 Expression of genes of proteins that comprise or interact with the renin-angiotensin system (RAS) in normal lung tissue (open bars n=49) and lung tumor tissue (solid bars, n =58) expressed as numeric values**. **Panel A** shows expression of genes encoding proteins that comprise the classical RAS. AGT encodes angiotensinogen, REN encodes renin, ACE encodes angiotensin-converting enzyme, AGTR1 encodes the AT_1_ Ang II receptor subtype, and AGTR2 encodes the AT_2_ Ang II receptor subtype. **Panel B** shows expression of genes encoding proteins that can alter the function of RAS but have many other functions. ATP6AP2 encodes the prorenin receptor (ATPase H(+)-transporting accessory protein 2), CMA1 encodes chymase, LNPEP encodes the Ang IV (AT_4_) receptor (insulin-regulated aminopeptidase), ENPEP encodes aminopeptidase A, and ANPEP encodes aminopeptidase N. One value for ANPEP was excluded from the data analysis because it was > 3 standard deviations apart from the rest of the dataset. **Panel C** shows expression of genes encoding proteins of the ACE2/Ang 1-7/MAS axis of the RAS which is an arm of the RAS that generally counteracts the actions of the classical RAS. ACE2 encodes angiotensin-converting enzyme-2, MME encodes neprilysin (neutral endopeptidase), PRCP encodes prolylcarboxypeptidase, PREP encodes prolylendopeptidase and MAS1 encodes MAS (Ang 1-7 receptor). * p<0.05 by unpaired t test with Welch’s correction for heterogeneity of variance and Sidak’s correction for multiple comparisons, ** p<0.01 by unpaired t test with Welch’s correction for heterogeneity of variance and Sidak’s correction for multiple comparisons.
